# Heart Rate Variability in Patients of Ankylosing Spondylitis: A Systematic Review and Meta-Analysis

**DOI:** 10.7759/cureus.51717

**Published:** 2024-01-05

**Authors:** Gaurav Sharma, Sagar Dholariya, Deepak Parchwani, Ragini Singh, Vinay Chitturi

**Affiliations:** 1 Physiology, All India Institute of Medical Sciences (AIIMS) Rajkot, Rajkot, IND; 2 Biochemistry, All India Institute of Medical Sciences (AIIMS) Rajkot, Rajkot, IND

**Keywords:** ankylosing spondylitis, non-invasive tool, autonomic nervous system, analysis, heart rate variability

## Abstract

Patients with ankylosing spondylitis (AS) have a significantly higher risk of cardiovascular morbidities. The participation of the autonomic nervous system (ANS) in AS is still unknown and inconclusive. Heart rate variability (HRV) is defined as the variability of the time interval between two consecutive heartbeats. This meta-analysis aims to detect the association of HRV and its various parameters with AS patients by comparing them to healthy controls.

Research literature was searched in PubMed, Embase, and Cochrane Library databases from inception to April 2022. The Review Manager 5 (RevMan) Version 5.4 software was used to analyze the data. In addition, the protocol of systematic review is registered in the PROSPERO database with ID CRD42022336484.

This study includes a total of nine case-control studies with a total of 923 patients; 409 with AS and 514 healthy controls. The root mean square of successive differences between normal heartbeats (RMSSD) [standardized mean difference (SMD); -0.47, 95% CI: -0.69 to -0.25, p < 0.0001], proportion of NN50 (pNN50) (SMD; -0.89, 95% CI: -1.74 to -0.04, p = 0.04) and HRV (SMD; -1.11, 95% CI: -1.53 to 0.69, P < 0.00001) were significantly low in AS cases compared to healthy controls. The HRV value was also significantly low in patients with high Bath ankylosing spondylitis disease activity (BASDAI) index (SMD: -1.45, 95% CI: -2.45 to -0.36, p < 0.009).

HRV (parasympathetic activity) was significantly lowered in AS patients compared to healthy controls.

## Introduction and background

Ankylosing spondylitis (AS) is a chronic autoimmune inflammatory disorder of the axial skeleton that mainly affects spinal joints, sacroiliac joints, and adjacent tendons and ligaments [1]. It clinically manifests as back pain, progressive stiffness of the spine, peripheral arthritis, and enthesopathy [2]. AS tends to develop more in young adults and occurs twice more often in males than females [3]. Cardiovascular morbidity and mortality are increased in AS patients, mainly due to the presence of chronic inflammation [4,5]. Diseases of the aortic root are the known cardiac complication of AS. At the same time, fibrosis-related conduction defects, arrhythmia, and left ventricular diastolic function abnormalities are rare complications of AS [6-8]. Furthermore, many studies reported that patients with AS were significantly associated with an increased risk of cardiovascular mortality [9,10] and many non-atherosclerotic cardiovascular morbidities [11].

Heart rate variability (HRV) is defined as the variability of the time interval between two consecutive heartbeats [12]. The more significant the difference in HRV, the greater the parasympathetic activity of the heart. A high HRV indicates that a person can continuously adapt to the microenvironment variations [13]. Consequently, a low HRV is associated with an increased risk of cardiovascular disease [14].

HRV was found to be lower in AS patients, and their values were negatively correlated with the blood level of C-reactive protein (CRP) and the score of Bath ankylosing spondylitis disease activity index (BASDAI) [15,16]. However, the non-significant result of HRV has also been reported in AS patients [17]. Thus, various studies reported inconclusive and conflicting results between AS and the autonomic nervous system [15,16,18]. Furthermore, the relationship between autonomic dysfunction and clinical characteristics, CRP, erythrocyte sedimentation rate (ESR), HLA B-27, and activity score of AS have not been explained comprehensively.

The autonomic nervous system (ANS) is vital in regulating cardiac functions. Several rheumatologic diseases, including AS, are characterized by the involvement of the central nervous system (CNS), peripheral nervous system (PNS), as well as autonomic nervous system (ANS) [19-22]. The involvement of the ANS has been evaluated earlier in AS by HRV and cardiovascular reflex tests. However, the role of the ANS in AS is still not fully understood, and results are inconclusive [23]. All parameters of HRV are influenced by the ANS and are primarily controlled by the PNS [24]. It is challenging to diagnose cardiac involvement in patients with AS because of aortic incompetence and bradycardia [25]. Even though standard cardiovascular reflex testing remains the gold standard [26], measuring HRV is the most reliable, simple, painless, repetitive, and non-invasive method of detecting autonomic cardiac dysfunction or autonomic neuropathy of the heart [27,28]. This meta-analysis aims to detect the association of HRV and its various parameters with the AS by comparing them to healthy controls.

## Review

Methods

Literature Search

We summarized all published studies that measured HRV in patients of AS and healthy controls. The research literature was searched in databases such as PubMed, Embase, and Cochrane Library, using the following MeSH keywords of the English language: ‘Ankylosing Spondylitis’ and (‘Heart Rate Variability or ‘HRV’) from inception to August 2022 (see Supplementary Table in the Appendix). Furthermore, the reference lists of all publications were searched manually to incorporate any additional studies that were not identified through the preliminary literature search. This study included observational case-control studies that measured HRV in patients with AS and healthy controls as a primary objective. In addition, studies that measured any non-linear HRV parameters or frequency and HRV times to assess autonomic cardiac regulation in AS patients were also included. The protocol of systematic review is registered in the PROSPERO database with ID CRD42022336484. The systematic review was performed as per the recommendations of the Preferred Reporting Items for Systematic Reviews and Meta-Analyses (PRISMA 2020) statement [29].

Ethical Considerations

Ethical approval was not taken for this study because the data used in this study has already been published, and there was no direct engagement of patients.

Extraction of Data

One author (SD) was involved in the literature search, article collection, and data extraction. Next, two authors (GS and RS) independently revised the abstracts and determined whether the article met the inclusion criteria for systematic review. When agreement on appropriateness could not be obtained, the disputed articles were reviewed by the other authors (DP and VC). All of the authors then reviewed the eligible articles. Finally, the following data were extracted from the selected articles: name of the authors, publication year, place of the study, sample size, and various parameters of the HRV and their association or correlation with other clinical and biochemical variables.

Assessment of the Quality

The quality assessment was performed according to the modified form of the Newcastle Ottawa Scale [30]. This scale includes three assessment domains: the selection of participants, comparability, and outcome procedures utilized in the included observational studies. Further, domains contain six subdomains, each receiving a score of one. Results with lower scores of ≤ 3 were interpreted as having a more significant potential for bias [31].

Parameters of HRV and Heart Rate Turbulence

HRV parameters measured in both the time domain and spectral domain were included in this study (Table 1). The following parameters were mainly reviewed in the time domain: RR intervals, root mean square of successive differences between normal heartbeats (RMSSD), standard deviations of the RR intervals (SDNN), and the percentage of adjacent NN intervals differ by more than 50 milliseconds (pNN50). SDNN is associated with low-frequency (LF) power, whereas pNN50 and RMSSD are related to high-frequency (HF) power, hence with parasympathetic activity. The following parameters were reviewed in the spectral domain: very low frequency (VLF; 0.003 to 0.04 Hz), Low Frequency (LF: 0.04 to 0.15 Hz), High Frequency (HF: 0.15 to 0.4 Hz), and LF/HF ratio. VLF reflects an alteration in renin-angiotensin system activity, the mechanism of thermoregulation, and the function of peripheral chemo-receptors. Furthermore, LF indicates the activity of both sympathetic and parasympathetic activity; HF indicates the parasympathetic activity of the Sinoatrial (SA) node, and LF/HF ratio represents the sympathovagal balance [32-34]. Heart rate turbulence (HRT) which includes turbulence onset (TO) and turbulence slope (TS), is also used to assess heart rate variability [35].

**Table 1:** Definition and importance of various parameters of HRV, electrocardiogram, and heart rate turbulence NN - Normal RR intervals

Parameters	Definition	Importance
HRV parameters		
SDNN (ms)	The standard deviation of NN intervals	Estimation of total HRV
SDSD (ms)	The standard deviation of differences between adjoining NN intervals	Indicates parasympathetic activity
RMSSD (ms)	The root mean square of successive differences between normal heartbeats	
pNN50 (%)	The percentage of adjoining NN intervals differs by more than 50 milliseconds	
SDNNI (ms) or SDNN index	Over 24 hours, the calculated mean of the 5-minute SD of NN intervals	Measurement of variability because of a short cycle of less than 5 minutes
VLF (ms)^2^	Very low-frequency power, 0.003 to 0.04 Hz	Indicates an activity of renin-angiotensin system activity, the mechanism of thermoregulation, and the function of peripheral chemo-receptors
LF (ms)^2^	Low-frequency power, 0.04 to 0.15 Hz	Indicates parasympathetic and sympathetic activity and baroreceptors functioning
LF (nU)	Normalized low-frequency power	Indicates sympathetic activity
HF (ms)^2^	High-frequency power, 0.15 to 0.4 Hz	Indicates parasympathetic activity and heart rate changes due to respiratory sinus arrhythmia
HF (nU)	Normalized high-frequency power	Indicates parasympathetic activity
LF/HF ratio	The ratio of low-frequency to high-frequency power	Indicates sympathovagal balance
TP (ms)^2^	Total power	Indicates variance of entire NN intervals
Heart rate turbulence		
TS or Turbulence Slope (ms/beat)	Associated with a higher risk of developing cardiovascular events	Indicates deceleration of sinus rhythm after premature ventricular contraction
TO or Turbulence Onset (%)	Percentage of variation in the two normal NN intervals following a premature ventricular contraction	Indicates acceleration of sinus rhythm after premature ventricular contraction

Data Synthesis and Statistical Analysis

This study was conducted to determine the HRV difference between AS cases and healthy controls. HRV parameters were reported in mean and standard deviation. The HRV parameters mentioned as the median and interquartile ranges were changed to their respective mean and standard deviation [36]. The standard mean differences (SMDs) with 95% confidence intervals were used to assess effect size in continuous data. The I2 statistic was used to calculate the extent of the inconsistency. The I2 values of 25%, 25-50%, and >50% were considered low, moderate, and a high degree of heterogeneity, respectively. In the absence of statistical heterogeneity, a fixed-effect model was employed. In contrast, a random-effect model was applied in the existence of statistical heterogeneity (defined by an I2 value > 50% or a chi-square p-value of < 0.05) [37]. As the number of selected studies was less than 10, a funnel plot to evaluate publication bias was not included. The Review Manager 5 (RevMan) version 5.4 software was used to evaluate all observed data [38].

Results

**Figure 1:**
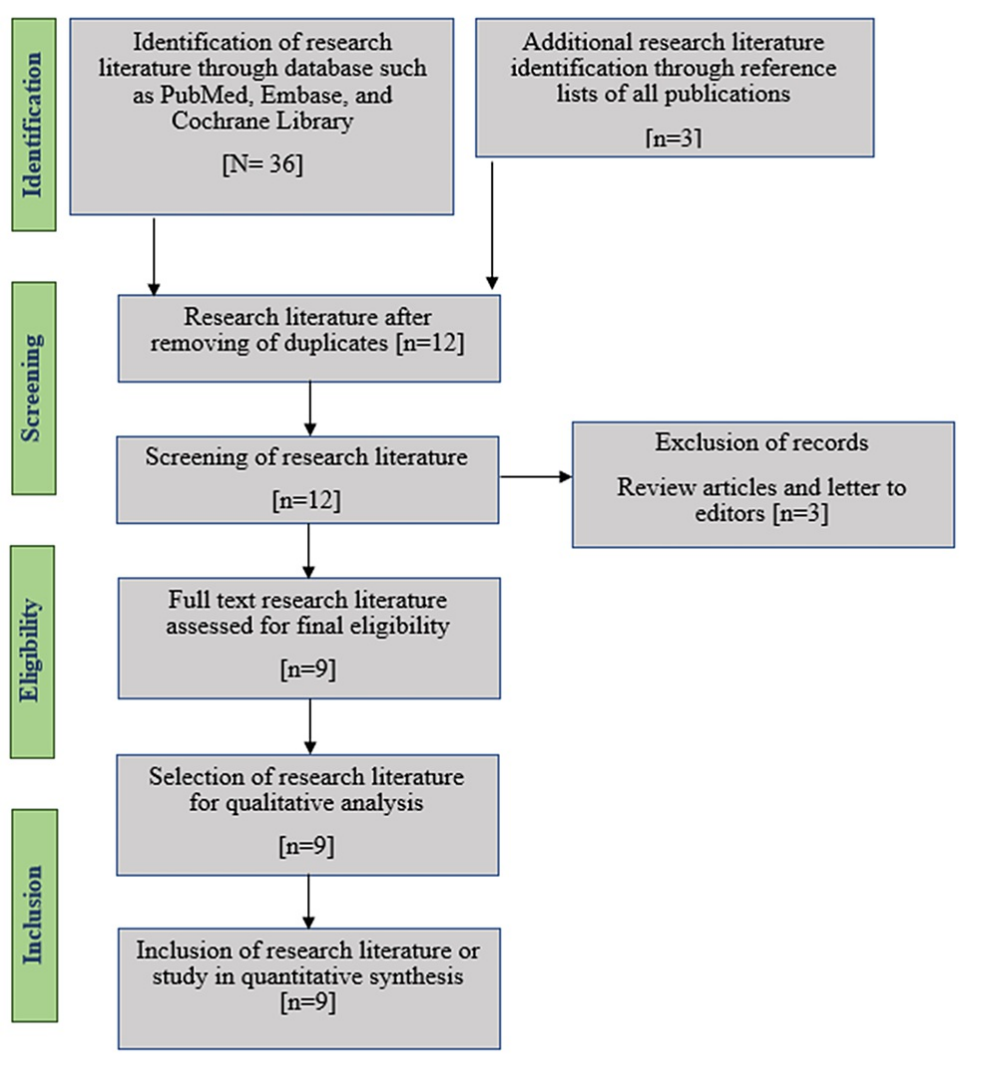
The flow chart of the PRISMA-recommended process for selecting research literature in the systematic review.

We primarily retrieved a total of thirty-nine studies from various databases. Finally, after excluding duplicates, case reports, letter to editors and case series, we included nine observational case-control studies for this systematic review and meta-analysis to reach a conclusion. Figure 1 represents the flow of the PRISMA-recommended process [29] for selecting the eligible studies.

A total of 923 participants were examined from the selected nine observational case-control studies that compared HRV parameters analysis in AS cases and healthy controls. Most of the included studies reported a low risk of bias (none of the studies reported a score of ≤ 3) according to the modified NOS scale, as shown in Table 2. Table 3 depicts the included studies with their place, sample size, method of HRV assessment, and main results or outcomes. The baseline characteristics of the included studies and the value of measured HRV parameters are shown in Table 4 and Table 5, respectively [16-18,23,25,39-42]. The methods used for HRV analysis and included HRV parameters were different among the included studies. In qualitative analysis, most studies have reported an overall decrease in measured HRV parameters except LF/HF ratio, which was elevated in AS [17,18,39]. Studies that measured similar HRV parameters were further included in the meta-analysis to derive the final outcome [16-18,23,25,40-42].

**Table 2:** Assessment of risk of bias by modified Newcastle-Ottawa scale 0 = No, 1 = Yes. Total score of less than or equal to 3 is a high risk of bias. AS - Ankylosing Spondylitis; HRV - Heart Rate Variability

Included studies	Selection of participants		Comparison			Outcome	Total score (High-risk of bias ≤ 3)
	Diagnosis of AS	Recruitment of patients and controls	Sex-matched	Age-matched	Exclusion criteria	Analysis of HRV and its parameter	
Borman et al., 2008 [16]	1	1	1	1	1	1	6
Yildirir et al., 2001 [17]	1	1	1	1	1	1	6
Kaya et al., 2010 [18]	1	1	1	1	1	1	6
Wei et al., 2016 [23]	1	1	1	1	1	1	6
Kazmierczak et al., 2008 [25]	0	1	1	1	1	1	5
Candemir et al., 2020 [39]	1	1	1	1	1	1	6
Gunes et al., 2009 [40]	1	1	1	1	1	1	6
Gunay et al., 2020 [41]	1	1	1	1	1	1	6
Poddubnyĭ et al., 2009 [42]	1	1	1	1	1	1	6

**Table 3:** Method of HRV assessment, observation, and main results of the included studies AS - Ankylosing Spondylitis; BASDAI - Bath Ankylosing Spondylitis Disease Activity Index; CRP - C-Reactive Protein; ECG - Electrocardiography; ESR - Erythrocyte Sedimentation Rate; HF - High Frequency; HRV - Heart Rate Variability; LF - Low Frequency; pNN50 - the division of the number of interval differences of successive NN intervals of more than 50 ms by the total number of NN intervals; RMSSD - Root mean square of successive differences between normal heartbeats; SDANN - Average standard deviation of the averages of all normal-to-normal R-R intervals; SDNN - Standard deviation of the normal-to-normal (NN) interval; SDSD - Standard deviation of successive RR interval differences.

Author of study	Place of study	Sample size	Method of assessment of HRV parameter	Observation and results
Borman et al., 2008 [16]	Turkey	Cases, n=20 Controls, n=20	Electromyograph	HRV, mean of R-R interval and heart rate response to standing were significantly low in cases of AS compared to controls. HRV parameters were significantly correlated with CRP and BASDAI index.
Yildirir et al., 2001 [17]	Turkey	Cases, n=94 Controls, n=49	ECG system-high resolution	There was no significant difference in HF, HF nU, LF, LF nU, and LF/HF ratio in patients of AS and healthy control groups.
Kaya et al., 2010 [18]	Turkey	Cases, n=28 Controls, n=30	24 h ambulatory electrocardiographic monitorization (AECG)	Decreased SDNN, RMSDD, SDANN and PNN50 Increased LF and LF/HF ratio in cases of AS compared to controls.
Wei et al., 2016 [23]	Taiwan	Cases, n=42 Controls, n=230	HRV analyzer	Decreased LF, LF in the normalized unit, and LF/HF ratio in patients of AS compared to healthy controls. There was a negative relationship between HF with CRP and ESR.
Kazmierczak et al., 2008 [25]	Poland	Cases, n=31 Controls, n=22	Holter monitoring and ECG	Decreased RMSSD and ultra-low frequency power in cases of AS compared to healthy subjects.
Candemir et al., 2020 [39]	Turkey	Cases, n=76 Controls, n=55	24-h Holter ECG recordings	Decreased LF, VLF, HF, SDNN, RMSSD, SDANN, and pNN50. Increased TS and TO in cases of AS compared to controls. Holter parameters were negatively correlated with the duration of AS.
Gunes et al., 2009 [40]	Turkey	Cases, n=35 Controls, n=55	24-h Holter ECG recordings	Decreased SDSD, RMSSD, and pNN50 in patients of AS compared to controls. HRV parameters were negatively correlated with isovolumetric relaxation and mitral inflow deceleration time.
Gunay et al., 2020 [41]	Turkey	Cases, n=32 Controls, n=30	ECG equipment	HRV and RRIV were significantly lower in AS patients with BASDAI index of ≥4.
Poddubnyĭ et al., 2009 [42]	Russia	Cases, n=51 Controls, n=23	ECG equipment	Decreased frequency and basic time of HRV parameters in patients of AS compared to controls. HRV parameters were negatively correlated with CRP, ESR, and fibrinogen.

**Table 4:** Baseline characteristics of the included studies BMI - Body mass Index; CRP - C-Reactive Protein; DBP - Diastolic Blood Pressure; M - Male; F - Female; SBP - Systolic Blood Pressure

Author of study	Study group	Age in years	Gender (M/F)	SBP (mmHg)	DBP (mmHg)	BMI in kg/m^2^	CRP in mg/dl	Duration of disease
Borman et al., 2008 [16]	Cases (n=20)	38.0 ± 8.5	15/5	Not mentioned	Not mentioned	25.3 ± 4.2	2.1 ± 2.0	9.75 ± 6.5
	Controls (n=20)	40 ± 9.8	14/6	Not mentioned	Not mentioned	25.5 ± 3.9	Not applicable	Not Applicable
Yildirir et al., 2001 [17]	Cases (n=94)	33.0 ± 11.0	47/47	Not mentioned	Not mentioned	Not mentioned	Not mentioned	5.5 ± 5.6 years
	Controls (n=49)	33.0 ± 8.0	23/26	Not mentioned	Not mentioned	Not mentioned	Not mentioned	Not Applicable
Kaya et al., 2010 [18]	Case (n=28)	28.7 ± 5.7	24/4	113.0 ± 4.2	73.1 ± 9.6	Not mentioned	Not mentioned	Not mentioned
	Control (n=30)	29.3 ± 5.8	26/4	114.8 ± 8.4	71.8 ± 8.0	Not mentioned	Not mentioned	Not Applicable
Wei et al., 2016 [23]	Case (n=42)	38.1 ± 12.3	32/10	Not mentioned	Not mentioned	23.6 ± 2.9	1.3 ± 2.6	8.3 ± 6.2
	Control (n=230)	38.0 ± 13.5	173/57	Not mentioned	Not mentioned	23.2 ± 4.1	Not mentioned	Not Applicable
Kazmierczak et al., 2008 [25]	Case (n=31)	50.5 ± 13.6	25/6	Not mentioned	Not mentioned	Not mentioned	Not mentioned	16.6 ± 9.7
	Control (n=22)	48.7 ± 12.6	17/5	Not mentioned	Not mentioned	Not mentioned	Not mentioned	Not Applicable
Candemir et al., 2020 [39]	Case (n=76)	38.8 ± 2.8	51/25	Not mentioned	Not mentioned	24.74 ± 3.21	0.86 ± 2.4	5 (2–9)
	Control (n=55)	36.5 ± 2.6	30/25	Not mentioned	Not mentioned	25.36 ± 3.81	0.6 ± 0.1	Not Applicable
Gunes et al., 2009 [40]	Case (n=35)	33.5 ± 9.4	23/12	121.8 ± 17.8	77.4 ± 9.9	25.0 ± 4.9	1.7 ± 0.3	90.6 ± 69.9
	Control (n=25)	36.9 ± 10.4	17/8	119.4 ± 10.6	77.6 ± 7.2	23.2 ± 2.3	0.3 ± 0.08	Not Applicable
Gunay et al., 2020 [41]	Case (n=32)	38.8 ± 9.3	18/14	Not mentioned	Not mentioned	Not mentioned	Not mentioned	11.7 ± 9.0
	Control (n=30)	37.5 ± 8.9	16/14	Not mentioned	Not mentioned	Not mentioned	Not mentioned	Not Applicable
Poddubnyĭ et al., 2009 [42]	Case (n=51)	35.4 ± 7.3	51/0	Not mentioned	Not mentioned	Not mentioned	Not mentioned	Not mentioned
	Control (n=23)	35.7 ± 11.5	23/0	Not mentioned	Not mentioned	Not mentioned	Not mentioned	Not Applicable

**Table 5:** Values of different parameters of HRV in AS cases and healthy controls LF - Low Frequency; HRV - Heart Rate Variability; HF - High Frequency; pNN50 - percentage of beats in which the change in successive normal sinus (NN) intervals exceeds 50 ms; SDNN - Standard deviation of the normal-to-normal (NN) interval; RMSSD - Root mean square of successive differences between normal heartbeats.

HRV parameter in Mean ± SD	Study group	Yildirir et al., 2001 [17]	Kaya et al., 2010 [18]	Wei et al., 2016 [23]	Kazmierczak et al., 2008 [25]	Candemir et al., 2020 [38]	Gunes et al., 2009 [39]
SDNN (ms)	AS Cases	Not mentioned	133.5 ± 46.9	Not mentioned	154.0 ± 35.0	146.6 ± 38.3	158.4 ± 40.0
	Controls	Not mentioned	173.9 ± 50.5	Not mentioned	162.0 ± 45.0	157.2 ± 17.5	140.8 ± 56.0
RMSSD (ms)	AS Cases	Not mentioned	37.3 ± 24.9	Not mentioned	55.0 ± 32.0	36.6 ± 12.05	42.7 ± 18.4
	Controls	Not mentioned	48.9 ± 21.2	Not mentioned	74.0 ± 29.0	40.98 ± 7.4	58.8 ± 42.6
pNN50 (%)	AS Cases	Not mentioned	11.1 ± 8.4	Not mentioned	Not mentioned	13.6 ± 1.9	12.0 ± 8.6
	Controls	Not mentioned	16.4 ± 10.3	Not mentioned	Not mentioned	16.4 ± 1.2	16.2 ± 11.1
LF (ms)^2^	AS Cases	2.7 ± 0.4	31.6 ± 7.9	5.4 ± 1.2	941.0 ± 585.0	24.2 ± 2.3	Not mentioned
	Controls	2.8 ± 0.3	20.2 ± 5.1	5.8 ± 1.1	1024.0 ± 589.0	27.8 ± 1.7	Not mentioned
HF (ms)^2^	AS Cases	2.1 ± 0.6	10.2 ± 3.1	5.46 ± 1.0	489.0 ± 427.0	15.0 ± 1.5	Not mentioned
	Controls	2.1 ± 0.6	10.5 ± 3.4	5.31 ± 1.1	505.0 ± 277.0	17.2 ± 1.2	Not mentioned
LF/HF ratio	AS Cases	0.6 ± 0.4	3.8 ± 1.7	0.005 ± 0.4	2.0 ± 1.0	1.7 ± 0.4	Not mentioned
	Controls	0.5 ± 0.4	2.7 ± 1.1	0.55 ± 0.7	2.0 ± 0.6	1.5 ± 0.2	Not mentioned

Assessment of HRV Parameters

**Figure 2:**
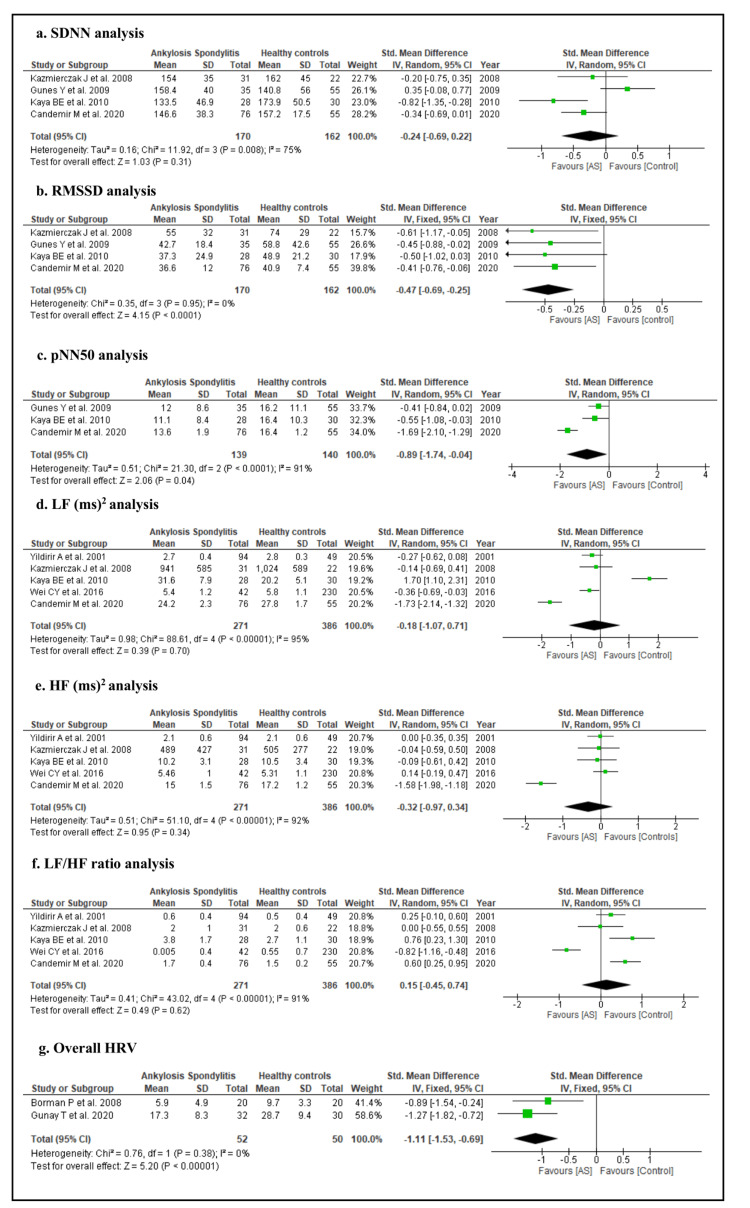
Analysis of HRV and its parameter in AS patients and healthy controls Borman et al., 2008 [16]; Candemir et al., 2020 [39]; Gunay et al., 2020 [41]; Gunes et al., 2009 [40]; Kaya et al., 2010 [18]; Kazmierczak et al., 2008 [25]; Wei et al., 2016 [23]; Yildirir et al., 2001 [17].

A total of four studies reported SDNN and RMSSD analysis. SDNN was comparable in AS patients and healthy controls as the effect size was not significant in a random-effect model of analysis (SMD: -0.24, 95% CI: -0.69 to 0.22, Z = 1.03, p = 0.31) as shown in Figure 2a. But, RMSSD was significantly low in AS cases compared to healthy controls (SMD: -0.47, 95% CI: -0.69 to 0.25, Z = 4.15, p <0.0001) in a fixed-effect model of analysis as shown in Figure 2b. Similarly, pNN50 was also significantly low in AS patients compared to healthy controls in a random-effect model of analysis [SMD: -0.89, 95% CI: -1.74 to -0.04, Z = 2.06, p < 0.04), Figure 2c]. Furthermore, LF [SMD: -0.18, 95% CI: -1.07 to 0.71, Z = 0.39, p < 0.70), Figure 2d], HF [SMD: -0.32, 95% CI: -0.97 to 0.34, Z = 0.95, p = 0.34), Figure 2e], and LF/HF ratio [SMD: 0.15, 95% CI: -1.45 to 0.74, Z = 0.49, p = 0.62), Figure 2f] were not significantly different in AS patients and healthy controls.

**Figure 3:**
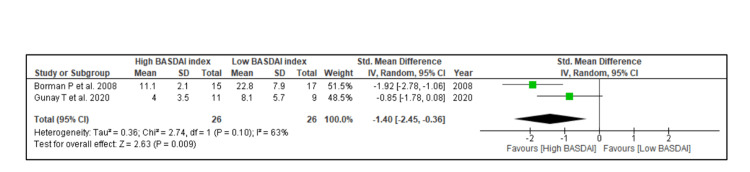
HRV analysis in relation to BASDAI index in AS patients Borman et al., 2008 [16]; Gunay et al., 2020 [41].

The overall HRV value was assessed only by two included studies in AS. It was found that the overall HRV value was significantly low in AS cases compared to healthy controls in a fixed-effect model of analysis (SMD: -1.11, 95% CI: -1.53 to -0.69, Z = 5.20, p < 0.00001), Figure 2g]. In addition, HRV analysis concerning the BASDAI index in AS patients was reported in only two studies. It was found that overall HRV value was significantly low in patients with a high BASDAI index (SMD: -1.45, 95% CI: -2.45 to -0.36, Z = 2.83, p < 0.009) as shown in Figure 3.

Discussion

According to our knowledge and various database searches, this is the first meta-analysis to evaluate HRV parameters in AS patients compared to healthy controls. We observed an overall decrease in HRV parameters in patients with AS compared to healthy controls, revealing the consequence of AS on the cardiovascular system by affecting ANS. It was found that ANS dysfunction in patients with AS is predominantly associated with PNS, and this dysfunction was associated with the severity or activity of AS [16,41]. We reported high heterogeneity across the included observational studies during the meta-analysis of HRV parameters. Hence, we measured SMD in a random-effect model for the studies with a heterogeneity of >50.0% during assessing specific HRV parameters.

The most commonly evaluated parameters were SDNN, RMSSD, pNN50, LF, HF, LF/HF ratio, and HRV value in the included studies. We found significant low value of RMSSD in AS patients compared to healthy controls (SMD: -0.47, 95% CI: -0.69 to 0.25, Z = 4.15, p < 0.0001). RMSSD is the main time domain that indicates beta-to-beat difference and is found to be associated with heart rhythm by affecting vagal nerve changes. Furthermore, the PNS significantly impacts the RMSSD compared to the SDNN [12]. Similarly, pNN50 was reported low in AS patients compared to healthy control in this meta-analysis (SMD: -0.89, 95% CI: -1.74 to -0.04, Z = 2.06, p < 0.04). The pNN50 also indicated PNS activity, and its value was correlated with HF and SDNN [12]. In addition, the HRV value was also significantly low in AS patients compared to healthy controls (SMD: -1.11, 95% CI: -1.53 to -0.69, Z = 5.20, p < 0.00001). Thus, reduced RMSSD, pNN50, and HRV values suggest that AS might cause a significant decrease in parasympathetic activity or vagal regulation of the heart rate.

But, the LF/HF ratio was elevated in patients with AS compared to controls (SMD: 0.15, 95% CI: -0.45 to 0.74). It might be due to the alteration of the sympathetic system in response to sympathovagal balance [18]. The low HRV was significantly associated with the AS patients with high BASDAI scores (SMD: -1.45, 95% CI: -2.45 to -0.36, Z = 2.83, p < 0.009). It suggests the association of parasympathetic dysfunction with the severity and active course of the AS. Most of the included studies have not reported a significant association of HRV parameters according to age and gender distribution. Hence, subgroup analysis of HRV concerning age and gender distribution was not performed. Similarly, subgroup analysis of HRV with CRP, erythrocyte sedimentation rate, and disease duration was not conducted due to the unavailability of required data in the included studies.

The impairment of the ANS in AS supports the hypothesis that an excessive inflammatory response is generated in many inflammatory disorders and autoimmune disorders due to the disturbance of the cholinergic pathway for anti-inflammation. This ANS impairment was mainly attributed to subsequent consequences of inflamed nerves resulting in inhibited nerve conduction [43,44]. In support of this view, various studies have reported deranged autonomic functions in diseases like rheumatoid arthritis, Behcet, lupus, and other inflammatory diseases, including Crohn’s disease [45,46]. Another possible explanation for deranged autonomic functions can be explained by the theory of cytokines, where it is suggested that overproduction and expression of various cytokines in autoimmune diseases, including ankylosing spondylitis, may lead to changes in HRV [47,48]. Furthermore, the systematic inflammation in AS seriously influences the autonomic regulation of cardiac function and decreases parasympathetic activity. Therefore, subnormal HRV parameters might contribute to the development of cardiovascular disease in AS.

Limitations and future perspectives

Firstly, one of the most important limitations is that different techniques or modalities were used to measure HRV parameters. Secondly, the cross-sectional design of the included studies with limited patients is the main limitation of this study. Due to the lack of prospective design, altered HRV parameters could not determine the actual prognostic significance. In addition, the limited sample size of study participants, duration and activity of AS, position of patients, and time of assessment may affect the values of HRV parameters.

The RMSSD and pNN50 accurately identify the parasympathetic activity and also correlate the survival in the presence of cardiac insufficiency. Considering the prognostic relevance of HRV, individuals with AS should be actively monitored for unfavorable cardiovascular events by conducting the prospective study. HRV should be recorded as per the guidelines laid down by the Task Force of The European Society of Cardiology and The North American Society of Pacing and Electrophysiology [49].

The Next-generation HRV (Next-gen HRV) integrates the parasympathetic, sympathetic, and output of Bayevsky’s tension index [50]. Next-gen HRV or immune autonomics could provide a novel approach to combat how the ANS stress level seems to exaggerate AS and possibly other autoimmune disorders. Next-gen HRV records ECG at a frequency of 8000 Hz, which minimizes inaccuracy in heartbeat separation and estimates the time across beats. Thus, HRV has progressed in hardware, software, and implementations to provide a dramatic transformation in integrating ANS stress response as a crucial personalized, new therapeutic component to deliver superior treatment for AS and other rheumatic disorders.

## Conclusions

HRV parameters were significantly disturbed in the patients of AS, indicating impairment of the cardiac autonomic nervous system. This meta-analysis stated that there is considerable evidence for an overall decrease in HRV in patients of AS. Therefore, HRV analysis can be used as a non-invasive tool to access and monitor disease activity or severity in patients of AS.

## Appendices

**Table 6:** Literature search strategy

Search strategy	Keywords
PubMed Search strategy	Slab 1: Ankylosing spondylitis #1 "spondylitis, ankylosing"[MeSH Terms] OR ("spondylitis"[All Fields] AND "ankylosing"[All Fields]) OR "ankylosing spondylitis"[All Fields] OR ("ankylosing"[All Fields] AND "spondylitis"[All Fields]) Slab 2: Heart rate variability #2 (("heart rate"[MeSH Terms] OR ("heart"[All Fields] AND "rate"[All Fields]) OR "heart rate"[All Fields]) AND variability[All Fields]) OR HRV[All Fields] Slab 3: Autonomic nervous system #3 "autonomic nervous system"[MeSH Terms] OR ("autonomic"[All Fields] AND "nervous"[All Fields] AND "system"[All Fields]) OR "autonomic nervous system"[All Fields] Slab 4: #4 #1 AND #2 AND #3
Cochrane Library Search strategy	Slab 1: Ankylosing spondylitis #1 spondylitis, ankylosing OR spondylitis AND ankylosing OR ankylosing spondylitis OR ankylosing AND spondylitis Slab 2: Heart rate variability #2 heart rate variability or HRV Slab 3: Autonomic nervous system #3 autonomic nervous system Slab 4: #4 #1 AND #2 AND #3
Embase Search strategy	Slab 1: Ankylosing spondylitis #1 ((ankylosis or ankylosing or ankylotic or ankylosans or ankylopoietic or rheumatoid or ankylopoietica) adj2 (spondyloarthrit or spondylarthritis or spondilitis or spondylarthrosis or or spondylitis or spine or spinal or vertebral)).tw,kw. Slab 2: Heart rate variability #2 (Heart rate variability:ti,ab OR HRV:ti,ab) Slab 3: Autonomic nervous system #3 Autonomic nervous system/exp Slab 4: #4 #1 AND #2 AND #3
